# Serum concentration of Selenium in healthy individuals living in Tehran

**DOI:** 10.1186/1475-2891-4-32

**Published:** 2005-11-14

**Authors:** R Safaralizadeh, GA Kardar, Z Pourpak, M Moin, A Zare, S Teimourian

**Affiliations:** 1 Immunology, Asthma & Allergy Research Institute, Children Medical Center, Tehran University of Medical Sciences, Tehran, P.O. Box: 14185-938, I.R.Iran

**Keywords:** selenium, serum, Tehran inhabitants

## Abstract

**Objective:**

To investigate whether daily diet provides adequate selenium intake in healthy men and women living in Tehran, Iran.

**Method:**

Serum level of selenium was determined in 184 healthy individuals of both genders. The samples were divided into two age groups, adults and children, for analysis. The serum level of selenium was determined using hydride generation and flame atomic absorption spectroscopy.

**Results:**

The mean and standard deviation of serum selenium levels in children (1–16 years) was 84.3 ± 11 μg/l and there was no significant difference between genders in this group. In adults (older than 16 years) the mean serum selenium level was 100.6 ± 13 SD μg/l; among women the mean was 93.9 ± 14 SD μg/l and among men it was 102.2 ± 12 SD μg/l. The mean selenium level in men was higher than in women and data analysis showed a significant difference between them (p < 0.005). There was also a positive correlation between higher selenium serum concentration and age in men (P < 0.001). Daily intake of selenium in men and women was calculated to be 67 μg and 62.1 μg respectively.

**Conclusion:**

Our results show that the serum concentration of selenium in an Iranian population is similar to other nationalities in the Middle East, particularly Saudi Arabia.

## Introduction

Selenium (Se) plays a key role in the maintenance of normal health in human populations [1]. The cellular biochemistry of Selenium involves the expression of a variety of selenoproteins. Selenium is part of the active site of glutathione peroxidase (GSH-Px), an antioxidant enzyme [2]. It has been demonstrated that, when taken as a supplement, Selenium modulates the cellular response to oxidative stress, inducing a faster restoration of the endogenous antioxidative defense system against the production of reactive oxygen species [3]. Glutathion peroxidase controls the interacellular level of hydrogen peroxide, reducing the formation of reactive oxygen species that can induce lipid peroxidations with consequent damage to the cellular membranes [4]. Epidemiological studies suggest a low intake of Selenium might predispose an individual to an increased incidence of cardiovascular disorders [5]. There is increasing evidence that selenium deficiency may have several serious short- and long-term medical implications, including impaired immune response, or even cancer [6]. An experimental study has shown that an increase in selenium level is associated with decreased cancer mortality [7]. The recommended dietary allowance of Selenium in the USA is 55 μg/day for women and 70 μg/day for men. In some regions of the world such as Finland, New Zealand, the East coast of the United States of America and China the content of Selenium in soil is remarkably low [4]. Therefore Selenium levels in the serum of populations throughout the world vary from 41.7 μg/l in Finland to 158.2 μg/l in Canada [8]. There is currently no information of selenium intake and serum levels in the Iranian population. The aim of our study was therefore to evaluate serum levels of Selenium to find out whether daily diet provides adequate selenium intake to maintain the health of men and women living in Tehran.

## Materials and methods

### 1. Subject selection

Serum samples were collected from 184 random inhabitants of Tehran. An informed consent was acquired, according to the guidelines from the Tehran university research ethic committee. Some of the samples were collected from excessive serum residues of blood taken from children who were referred for routine laboratory check up at the Children's Medical Center. All the samples were tested to rule out HIV, HBV and HCV contamination. Blood samples were left to coagulate spontaneously.

### 2. Determination of serum selenium

Blood samples (3 ml) were centrifuged at 3000 × g for 5 minutes. The clean serum was stored at -70°C until the time of analysis. All glassware and bottles used for the isolation of serum and for analysis were previously soaked in diluted nitric acid (10%) and rinsed thoroughly with deionized water. This procedure was followed in order to exclude the possibility of contamination with trace elements. Serum (500 μl) was aliquoted into a vessel-tube for mineralization with 3 ml of HNO_3_/HCLO_4 _(4:1 v/v). The temperature of this mixture was slowly increased to 175°C until fumes of HCLO_4 _appeared. The mixture was then heated according to the following (temperature/time) scheme: 175°C/60 min, 200°C/60 min and finally 250°C for 60 min. The mixture was then left to cool down to room temperature. HCL 6 N (10 ml) was added and heated to 170°C for 30 min to reduce the Se (VI) to Se (IV). After cooling to room temperature, Se concentration was determined using the hydride generation atomic absorption spectrometry (Atomic absorption spectrometer Shimadzu, AA-680). Sodium borohydride solution (3 g NaBH_4_, 1 g NaOH in 100 ml of mili-Q water) was used as a reducing agent. All samples and standards were analysed in duplicate. The accuracy of the procedure was evaluated by analyzing commercially available samples of lyophilized human serum seronorm™ trace element serum, level 1, M10181 indicating a recommended value of 81 μg/l, and seronorm™ trace element serum, level 2, NO0371 indicating a recommended value of 136 μg/l.

### 3. Statistical analysis

Kolmogorov-Smirnov tests were carried out for normal distribution. The reference range for serum selenium was determined as the 95% confidence interval (CI) of means. Differences in selenium concentration between the male and female populations were analyzed with the Mann-Whitney U-test. P-values of less than 0.05 were considered significant.

## Results

The studied individuals were all healthy, non-smokers. Volunteer medical history and physical examination ruled out the presence of current disease in the studied individuals. None of the individuals showed any digestive symptoms indicative of nutrient malabsorption. The mean and standard deviation of the individual selenium levels in children (age 1–16) was 84.2 ± 11 μg/l, which was 85.1 ± 10.8 μg/l among females and 83.7 ± 11.2 μg/l among males. The mean serum selenium level in adults (over 16 years) was 100.6 ± 12.9 μg/l, for adult women the mean was 93.9 ± 13.6 μg/l and for adult men was 102.1 ± 12.3 μg/l (Table 1). The accuracy and precision of the methods used for selenium analysis are summarized in Table 2.

**Table 1:** Age and sex of the studied individuals with corresponding selenium levels (μg/l)

Age	Sex	n	Min	Max	Mean ± SD	Reference Range (95% CI)
1–16 Y	F	22	66	105	85.0 ± 10.8	63–106
	M	32	58	105	83.7 ± 11.1	63–106
Over16 Y	F	24	74	125	93.9 ± 13.6	67–121
	M	106	75	134	102.1 ± 12.2	79–126

Y: Year, M: Male, F: Female, SD: Standard Deviation

P-value less than 0.05 is statistically significant

**Table 2:** Sensitivity and precision of the assay

**Material**	**Concentration**		**Accuracy **(%)	**Precision **R.S.D(%)
			
	Certified	Measured		
Seronorm™ Trace Element Serum (Level 1, M10181)	81 ± 1.5	80.4 ± 2.7	99.3	3.36
Seronorm™ Trace Element Serum (Level 2, NO0371)	136 ± 4.5	135 ± 3.8	99.2	2.81

R.S.D: relative standard deviation

## Discussion

Selenium is an essential mineral in human nutrition. Natural selenium present in the diet of humans is in the form of organic seleno-proteins such as selenomethionine and seleno-cysteine. Foods such as fish and whole grain cereals are especially rich in organic selenium compounds [11]. Selenium in cereals is primarily in the form of selenomethionine. This naturally occurring amino acid is the most important nutritional form of selenium.

Deficiencies of selenium contribute to the prevalence and severity of iodine deficiency disorders which are the most important and well-known global nutritional problems, primarily in less developed countries [12]. Iodine deficiency in childhood impairs neuromotor and intellectual development, with an average reduction in the intelligence quotient of 10 points [13]. Selenium is required in thyroid metabolism, converting inactive thyroid hormone into active thyroid hormone [14]. It has been shown that in goitrous children who are both Se and iodine deficient, major Se deficiency partially blunts thyroid response to iodine supplementation [15]. The mean serum Se level for healthy children (age 1–16) observed in this study was 84.2 ± 11 μg/l with no significant difference between sexes (p = 0.659). Table 3 shows the mean serum Se level of Iranian children compared to children from different countries.

**Table 3:** Comparison of the mean serum levels of selenium, among children from different countries (as mentioned previously [10])

**Country**	**Overall mean (μg/l)**	**Age range (years)**
Austria	48	1–15
Finland	58.5	1–15
Belgium	60	1–15
Germany	65.5	1–18
France	67	3–16
England	74.2	2–16
**Iran**	**84.3**	**1–16**
Japan	84.5	1–15
Slovenia	85	1–13
Turkey	89	1–16
US	106	1–18
Canada	126	1–9

The mean serum Se level for adults observed in this study was 100.6 ± 12.8 μg/l, which was similar to the one reported in a survey in Saudi Arabia [16]. In the Nutritional Prevention of Cancer (NPC) Trial [17], a Selenium level of 80 ng/mL is considered the minimum level of plasma selenium necessary in the bloodstream for maximum production of selenoproteins (glutathione peroxidases, thioredoxin reductase, etc.).

Our results show that in adults there is a significant difference between men and women (p < 0.005) with a higher concentration of selenium in men. This suggests a sex-linked hormonal influence over serum level of selenium. It has previously been shown that selenium is essential for spermatogenesis [18]. This trace element is present in the protein of the capsule surrounding the sperm mitochondria and may have a structural function [19]. Our data also show a positive correlation between a higher concentration of selenium in serum and age in men (P < 0.001).

Table 4 summaries the selenium serum levels in the Iranian population in comparison with different countries. It is higher than levels calculated for Finland and other countries where soil is poor in selenium content. By using the medium correlation factor (1.51) as introduced by Navarro et al., to estimate the daily intake of nutrients, the daily intake of Se was calculated as 62.19 μg in the female and 67 μg in the male populations [20].

**Table 4:** Comparison of the mean serum levels of selenium, among adults from different countries (as mentioned previously [9, 11])

**Country**	**Overall mean (μg/l)**
Finland (Helsinki)	41.7
New Zealand (Dunedin)	47.2
Brazil (Rio de Janeiro)	73.2
W. Germany (Mainz)	81.1
Sweden (lund)	85.0
Italy (Rome)	89.8
Japan (Hiroshima)	97.6
**Iran (Tehran)**	**100.3**
Saudi Arabia	102.5
US (Mort.Gr.)	110.2
England (Southampton)	115.7
Canada (Toronto)	158.3

Considering the American RDA, which recommends a daily Se intake of 50 μg for women and 70 μg for men, it seems that the normal Iranian diet has an adequate content of selenium for both genders.

Tehran, the capital of Iran, is located in the north of the country and has a population of approximately fifteen million people, representing a large proportion of the country's total population, estimated to be seventy five million people. The diversity of nourishment sources, regional variation and different ethnic diets makes it difficult to extend these results to the whole population.
